# First psychotic episode due to inmunosupresor medication

**DOI:** 10.1192/j.eurpsy.2023.2222

**Published:** 2023-07-19

**Authors:** A. Corrales, L. Unzue, L. Llovera, N. Cancelo

**Affiliations:** 1 Psychiatry, Navarra´s University Hospital, Pamplona

## Abstract

**Introduction:**

48-year-old man from Spain who lived with his wife until he got divorced 4 months before starting the follow up in Mental Health. The debut was in September 2021 with a hospitalization in the Brief-acute hospitalization unit due to florid psychotic clinic-

He consumed several drugs in his twenties (cocaine, marihuana and heroin IV) and was diagnosed of HIV at the age of 29. He abandoned the use of drugs after the diagnoses and keep good adherence to the antiretroviral treatment (Abacavir + Lamivudine +Efavirenz).

At the age of 46 (January 2020), he was successfully transplanted a kidney. Afterwards, he started taking inmunosupresor medication to avoid transplant rejection

At the few months of the transplant and the beginning of the inmusosupresor medication, the patient became more irascible with moments of remarkable disinhibition and progressive abandon of the work obligations.

In January of 2021, he got divorced after months of difficulties with his wife, married 28 years before, due to the mentioned problems as well as moments of bizarre and disorganized conducts and suspicion towards his wife with probable delusional jealousy. He, therefore, lost his job, hose and marriage and started taking drugs again after 17 years of abstinence.

He was hospitalized in e Brief Acute Inpatient Unit in September 2021 with distrustful and hypervigilant attitude- He was suffering from delusional ideation of harm and persecution with high distress and emotional repercussion. He also presented disorganized conduct and probable auditory hallucinations. He was positive to amphetamines and cocaine After 3 days without consuming; there was no remission of the clinic.

**Objectives:**

Discussing the association between the initiation of inmunsupresor medication and the beggining of psychotic clinic

**Methods:**

First psychotic episode (FEP) has a likely consequence of the initiation and maintenance of Tacrolimus -due to a kidney transplant- with the concomitant abuse of amphetamines and cocaine as a trigger factor.

The psychotic clinic progressively remitted in one week after the administration of 3 mg/day of risperidone.

The antiretroviral treatment was changed due to the poor adherence during the disorganization period. The tacrolimus was not removed because of the good response to the neuroleptic and the risk of transplant rejection

**Results:**

The patient started with prodromic symptoms of psychosis at the time he began with the inmunosupresor medication. Progresively, the psychotic clinic worsen wit the consequence of a biographical break with the consequence of a divorce, therlost of work and home and a drug relapse.

**Conclusions:**

There is evidence of the association between psychotic episodes in people with no psychiatric history and the inmunosupresor medication for the kidney transplant (Above all, tracolimus). This case remarks the need of an exhaustive medical anamnesis in the diagnosis of psychiatric pathologies.

**Disclosure of Interest:**

None Declared

